# Exposure to household pet cats and dogs in childhood and risk of subsequent diagnosis of schizophrenia or bipolar disorder

**DOI:** 10.1371/journal.pone.0225320

**Published:** 2019-12-02

**Authors:** Robert Yolken, Cassie Stallings, Andrea Origoni, Emily Katsafanas, Kevin Sweeney, Amalia Squire, Faith Dickerson

**Affiliations:** 1 Stanley Division of Developmental Neurovirology, Johns Hopkins School of Medicine, Baltimore, Maryland, United States of America; 2 Sheppard Pratt Health System, Baltimore, Maryland, United States of America; University of Taipei, TAIWAN

## Abstract

**Background:**

Serious psychiatric disorders such as schizophrenia and bipolar disorder have been associated with environmental exposures in early life. Contact with household pets such as cats and dogs can serve as a source of environmental exposure during these time periods.

**Methods:**

We investigated the relationship between exposure to a household pet cat or dog during the first 12 years of life and having a subsequent diagnosis of schizophrenia or bipolar disorder. These studies were performed in a cohort of 396 individuals with schizophrenia, 381 with bipolar disorder, and 594 controls. The hazards of developing schizophrenia or bipolar disorder associated with first exposure to a household pet cat or dog were calculated using Cox Proportional Hazard and multivariate logistic regression models including socio-demographic covariates.

**Results:**

We found that exposure to a household pet dog was associated with a significantly decreased hazard of having a subsequent diagnosis of schizophrenia (Hazard Ratio .75, p < .002) Furthermore, a significant decreased relative risk of schizophrenia was detected following exposure at birth and during the first years of life. There was no significant relationship between household exposure to a pet dog and bipolar disorder. There were no significant associations between exposure to a household pet cat and subsequent risk of either a schizophrenia or bipolar disorder diagnosis. However, there were trends towards an increased risk of both disorders at defined periods of exposure.

**Conclusions:**

Exposure to household pets during infancy and childhood may be associated with altered rates of development of psychiatric disorders in later life.

## Introduction

Schizophrenia and bipolar disorder are serious neuropsychiatric disorders with extensive morbidity and mortality worldwide. Family studies indicate a high rate of familial association for both disorders. Extensive genetic studies of both disorders have found a large number of genomic regions associated with increased risk; however few genes of large effect have been identified and there are substantial differences relating to genetic risk factors among different populations[1]. There has thus been an increasing interest in the identification of environmental factors which might interact with genetic factors to result in disease phenotypes. Of particular interest in this regard are environmental exposures in early life in light of the neurodevelopmental aspects of these disorders. Furthermore, household exposures during this time period are likely to be shared among family members and this might contribute to apparent familial associations of disease risk [2, 3].

The immune system has been identified as a pathway under the control of both genetic and environmental factors which may play an important role in the etiopathogenesis of schizophrenia and bipolar disorder.[4] Of particular importance is the role of the immune system in early life as a modulator of brain development, as documented by studies in humans and animal models [5]. In developed countries early life exposures to household pet animals such as cats and dogs have been identified as common environmental factors which can alter inflammation in infants and children. Mechanisms involved in immune modulation include regulation of the allergic response to allergens [6], exposure to zoonotic microbial agents [7], alterations in the microbiome [8], and the neuroendocrine effects of stress reduction associated with pet contact [9].

As part of our studies of gene-environmental interactions in psychiatric disorders, we collected data on household pet cat and pet dog exposure in a cohort of individuals with psychiatric disorders and controls along with a number of social and demographic factors. We employed this data set to investigate the relationship between early life exposure to a pet cat or pet dog and an adult diagnosis of schizophrenia or bipolar disorder.

## Methods

### Study population

The methods for the recruitment of the individuals with schizophrenia or bipolar disorder as well as control individuals without a psychiatric disorder have been previously described [10] [11] and are summarized as follows:

The inclusion criterion for individuals with schizophrenia was a current diagnosis of schizophrenia or schizoaffective disorder. The inclusion criterion for individuals with bipolar disorder was a DSM-IV diagnosis of bipolar disorder including bipolar I disorder, bipolar II disorder, or bipolar disorder not otherwise specified. Persons in both psychiatric groups also had an absence of a primary diagnosis of alcohol or substance use disorder over the past 3 months. Participants were recruited from inpatient, day hospital, and rehabilitation programs of Sheppard Pratt Health System and from affiliated psychiatric agencies located in or near Baltimore Md. USA. The diagnosis of each psychiatric participant was established by the research team including a board-certified psychiatrist and based on the Structured Clinical Interview for DSM-IV Axis 1 Disorders and available medical records. The individuals in the non-psychiatric control group were recruited from posted announcements at local institutions in the same geographic region as the psychiatric participants and were screened using the Structured Clinical Interview for DSM-4 or DSM-5 to rule out the presence of a current or past psychiatric disorder employing previously described procedures [12], The control group was drawn from the same geographic area as the individuals with psychiatric disorders. All participants met the following additional criteria: age 18–65; proficient in English; absence of any history of intravenous substance abuse; absence of mental retardation; absence of HIV infection; absence of serious medical disorder that would affect cognitive functioning.

#### Socio-Demographic Factors

Race/ethnicity was determined by self-report. For the purposes of analyses the individuals were divided into the following groups: White, African American, Asian, Hispanic, Other.

The highest level of parental education was used as a proxy marker for socioeconomic status during childhood. The highest level of maternal education was used if this information was available. Paternal education was substituted for maternal level of education for individuals on whom only data relating to paternal education was available. Individuals on whom neither maternal nor paternal education were available were excluded from the analysis. Place of birth was determined by self-report. Place of birth was divided into the following categories for the purpose of analysis: Baltimore City Maryland urban area; other locations in the state of Maryland USA; other locations in the United States and Canada; locations outside of the United States and Canada.

### Exposure to a household pet cat or dog

Individuals were queried in terms of whether they had a pet cat and/ or pet dog in their household during their lifetime and, if so, when was the first time and the most recent time they had a pet cat or a pet dog. The age of the first household pet cat or first household dog was used as an indicator of first exposure to the indicated household pet. An individual reporting that a household pet cat or pet dog was present at their time of birth was considered to be exposed to that household pet since birth.

### Statistical analyses

The relationship between the age of first household pet exposure during the first 12 years of life and psychiatric diagnosis was defined using Cox proportional hazard models. These models employed the diagnostic groups of schizophrenia and bipolar disorder as the outcome variables and were compared to the controls without a psychiatric disorder. An individual who reported a household pet cat or pet dog at birth was assigned an exposure age for that pet of 0.1 years of age for the purposes of analysis. Age, gender, race/ethnicity, parental education and place of birth defined as above were employed as covariates. These covariates were selected based on differences among the groups as shown in Table 1 as well as previously described demographic variables associated with pet exposure during infancy and childhood. Cox proportionate hazards models were constructed employing both the entire study population as well as populations censored for prior exposure to the other pet. For these analyses a population of individuals censored for prior exposure to a pet cat was employed for analyses of exposure to a pet dog and a population of individuals censored for prior exposure to a pet dog was employed for analyses of exposure to a pet cat. The age distributions of the censored populations by clinical diagnosis is depicted in S1 and S2 Tables. A detailed description of the data employed for the statistical analyses is presented in S3 Table.

**Table 1:** Demographic characteristics of the study population.

		Schizophrenia	Bipolar Disorder	Control
		N = 396	N = 381	N = 594
Age Mean (SD)		37.6 (13.5)	36.7 (13)	32.7 (11.4)
Parental Education		13.1 (2.6)	13.5 (2.9)	13.7 (2.7)
Gender (Female)	Number	126	259	374
	%	31.8	68.08	63
Race/Ethnicity				
White	Number	184	281	332
	%	46.5	73.8	55.9
African American	Number	196	75	186
	%	49.5	19.7	31.3
Asian	Number	5	13	51
	%	1.3	3.4	8.6
Hispanic	Number	6	1	8
	%	1.5	0.3	1.4
Other	Number	5	11	17
	%	1.3	2.9	2.9
Place of Birth				
Baltimore City	Number	219	178	257
	%	55.3	46.7	43.3
Rest of Maryland	Number	67	71	61
	%	16.9	18.6	10.3
Rest of United States and Canada	Number	88	111	222
	%	22.2	29.1	37.4
Outside of United States and Canada	Number	22	21	54
	%	5.6	5.5	9.1

The timing of relationship between the age group of exposure to the first household pet cat or dog and psychiatric diagnosis was further explored using multivariate logistic regression models. For these analyses the following age ranges were selected: birth, the period immediately after birth through age 3, ages 4–5, ages 6–8, and ages 9–12 inclusive. Relative risks of having a diagnosis of schizophrenia or bipolar disorder as compared to controls were calculated for all of these groups in comparison to the individuals not exposed to the indicated household pet before the 13^th^ birthday employing age, gender, race/ethnicity parental education, and place of birth as covariates. These analyses were performed both using the entire cohort as well as subsets censored for the opposite pet as described above.

The analyses involved 2 pets and 2 clinical diagnoses. Hence an alpha of p< = .0125 (.05/4) was employed as an indication of statistical significance. A value of .05< = p >.0125 was considered to indicate a trend level of significance.

### Ethical approval

The studies were approved by the Institutional Review Boards of the Sheppard Pratt Health System and the Johns Hopkins Medical Institutions following established guidelines to protect participants based on the Declaration of Helsinki. All participants provided written informed consent after the study procedures were explained.

## Results

The study population consisted of 1371 individuals, 396 of whom had a diagnosis of schizophrenia or schizoaffective disorder, 381 of whom had a diagnosis of bipolar disorder, and 594 control individuals without a history of a psychiatric disorder. Demographic and clinical variables relating to the study cohort are presented in Table 1.

The age range of first exposure to a household pet dog and household pet cat are depicted in Tables 2 and 3. Overall 220 (55.7%) of the individuals with schizophrenia were exposed to a pet dog before the 13th birthday as compared to 248 (65.2%) of the individuals with bipolar disorder and 389 (62.1%) of the control individuals. In terms of household pet cats, 139 (35.1%) of the individuals with schizophrenia were exposed to a pet cat before the 13th birthday as compared to 157 (41.2%) of the individuals with bipolar disorder and 206 (34.7%) of the control individuals.

**Table 2:** Age group of exposure to first pet dog by diagnostic group.

	Diagnostic Group	Schizophrenia	Bipolar Disorder	Control
		N = 396	N = 381	N = 594
**Age of First Pet Dog**				
Present at Birth	Number	32	71	100
	%	8.08	18.64	16.84
After Birth through Age 3	Number	17	34	46
	%	4.29	8.92	7.74
Age 4–5	Number	48	50	63
	%	12.12	13.12	10.61
Age 6–8	Number	69	54	83
	%	17.42	14.17	13.97
Age 9–12	Number	54	39	77
	%	13.64	10.24	12.96
Any Before age 13	Number	220	248	369
	%	55.55	65.09	62.12
None before Age 13	Number	176	133	225
	%	44.44	34.91	37.88

**Table 3:** Age group of exposure to first pet cat by diagnostic group.

	Diagnostic Group	Schizophrenia	Bipolar Disorder	Control
		N = 396	N = 381	N = 594
**Age of First Pet Cat**				
Present at Birth	Number	37	47	70
	%	9.34	12.34	11.78
After Birth through Age 3	Number	8	21	17
	%	2.02	5.51	2.86
Age 4–5	Number	24	27	33
	%	6.06	7.09	5.56
Age 6–8	Number	35	28	50
	%	8.84	7.35	8.42
Age 9–12	Number	35	34	36
	%	8.84	8.92	6.06
Any Before age 13	Number	139	157	206
	%	35.1	41.21	34.68
None before Age 13	Number	257	224	388
	%	64.9	58.79	65.32

**Fig 1:**
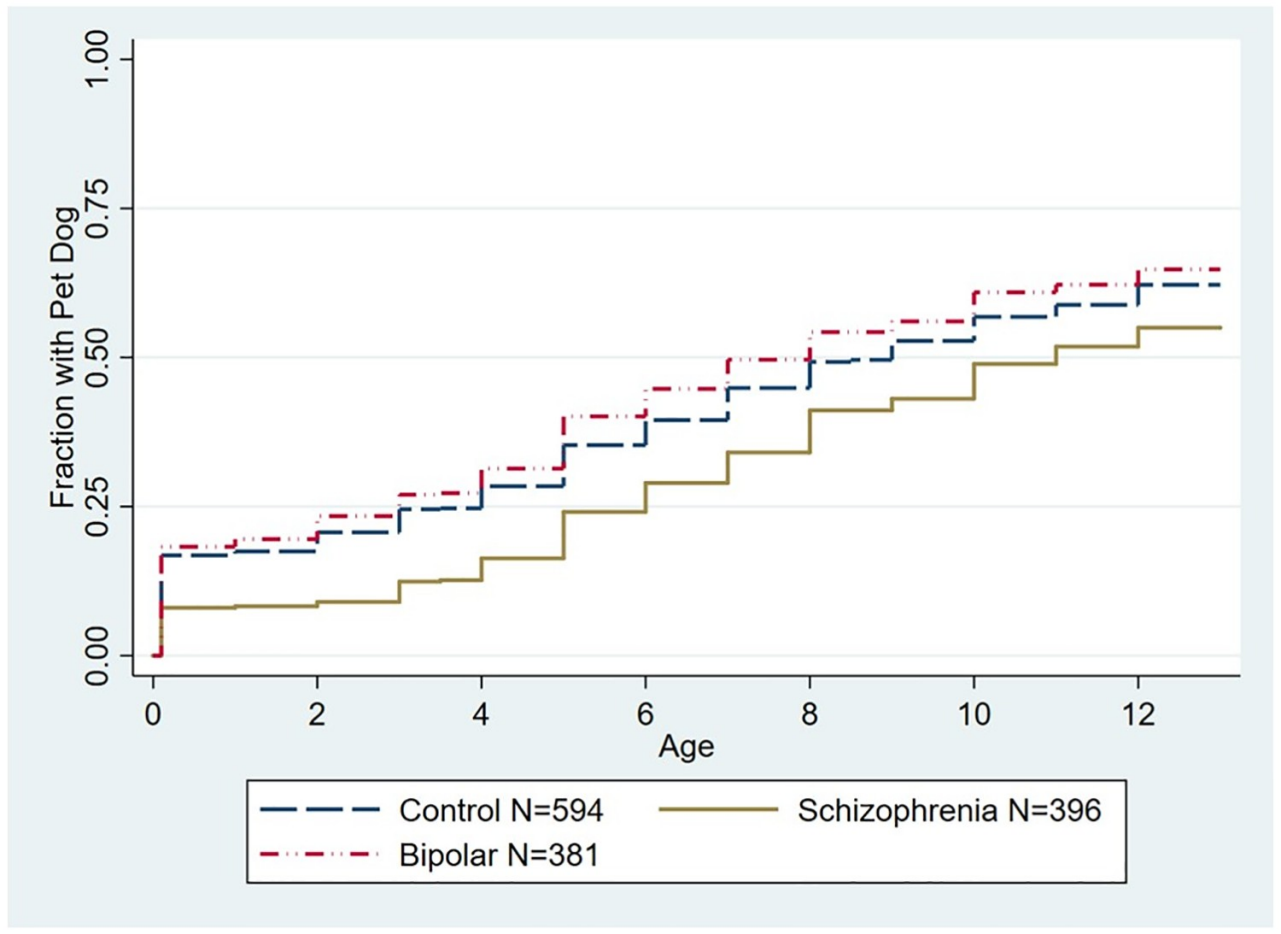
Kaplan Meier plot depicting accumulated proportion of exposure to first household pet dog stratified by diagnostic group. The group of individuals with schizophrenia differed from the control group (Hazard Ratio (HR) = .75 95% CI .63-.90, p < .002, adjusted for age, gender, race/ethnicity, parental education, and place of birth). The group of individuals with bipolar disorder did not differ significantly from the control group.

**Fig 2:**
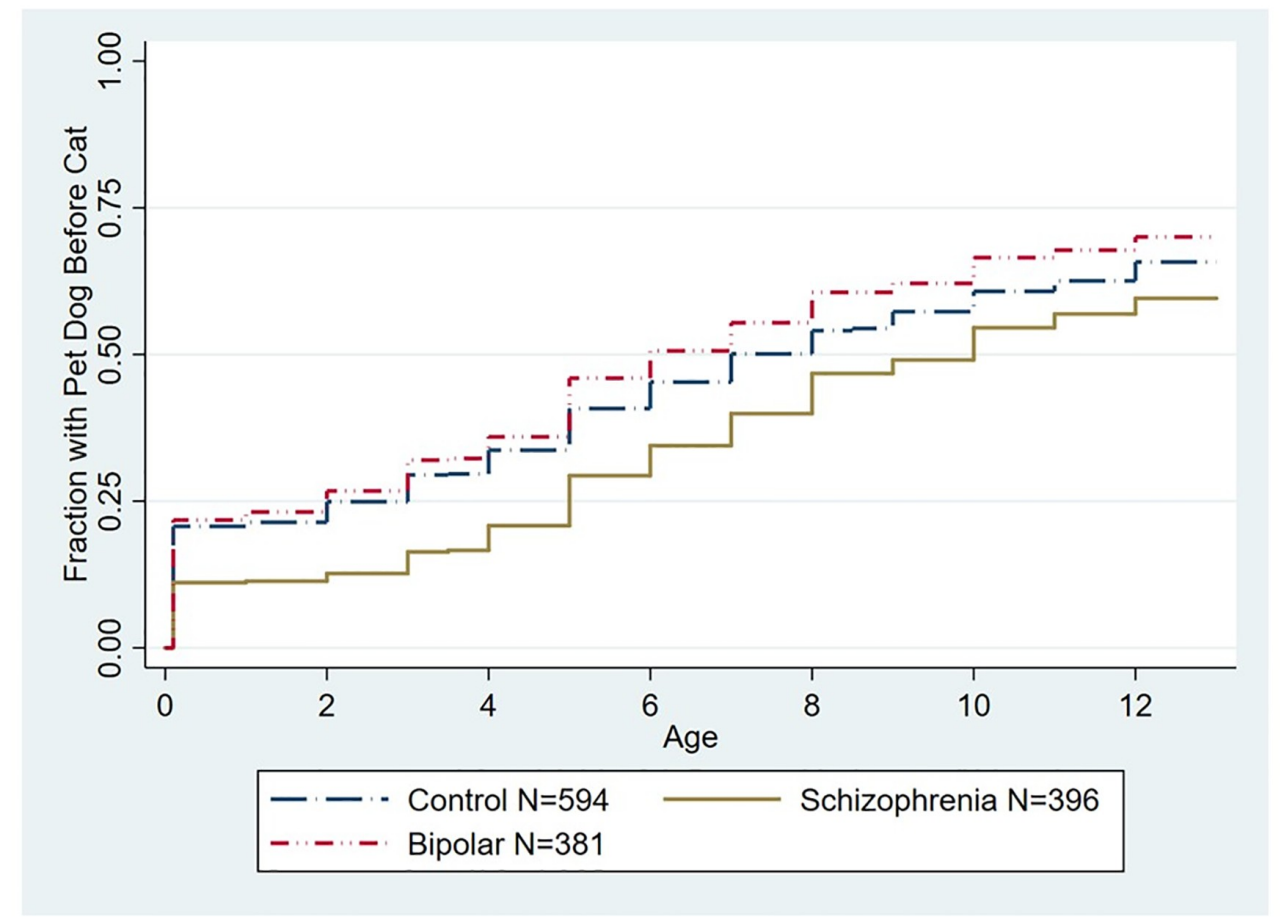
Kaplan Meier plot depicting accumulated proportion of exposure to first household pet dog stratified by diagnostic group censored for prior exposure to a household pet cat. The age distribution of the censored population is shown in S1 Table. The group of individuals with a schizophrenia diagnosis differed from the control group (HR .76, 95% CI .63 -.91, p < .002 adjusted for age, gender, race/ethnicity, parental education, and place of birth). The group of individuals with bipolar disorder did not differ significantly from the control group. The numbers shown indicate the total population including those who were censored.

The relationships between a psychiatric diagnosis and the age of first household pet dog or pet cat before the 13th birthday were analyzed using Cox proportional hazard models. As depicted in Fig 1, the time of the first household pet dog before the 13th birthday was associated with a significantly decreased hazard of having a schizophrenia diagnosis as compared to controls (Hazard Ratio (HR) = .75 95% CI .63-.90, p < .002, adjusted for age, gender, race/ethnicity, parental education, and place of birth). As depicted in Fig 2, we also examined models in which individuals who had a household pet cat prior to a household pet dog were censored from the model. In this model the time of the first household pet dog before the 13th birthday remained associated with a significantly decreased hazard of having a schizophrenia diagnosis (HR .76, 95% CI .63 -.91, p < .002 adjusted for age, gender, race/ethnicity, parental education, and place of birth). The age of first household pet dog was not associated with an altered hazard of diagnosis of bipolar disorder when considering the entire population (Fig 1). Similarly the age of first household pet dog was not associated with an altered hazard of diagnosis of bipolar disorder when examining the subset of the population censored for prior exposure to a pet cat (Fig 2).

**Fig 3:**
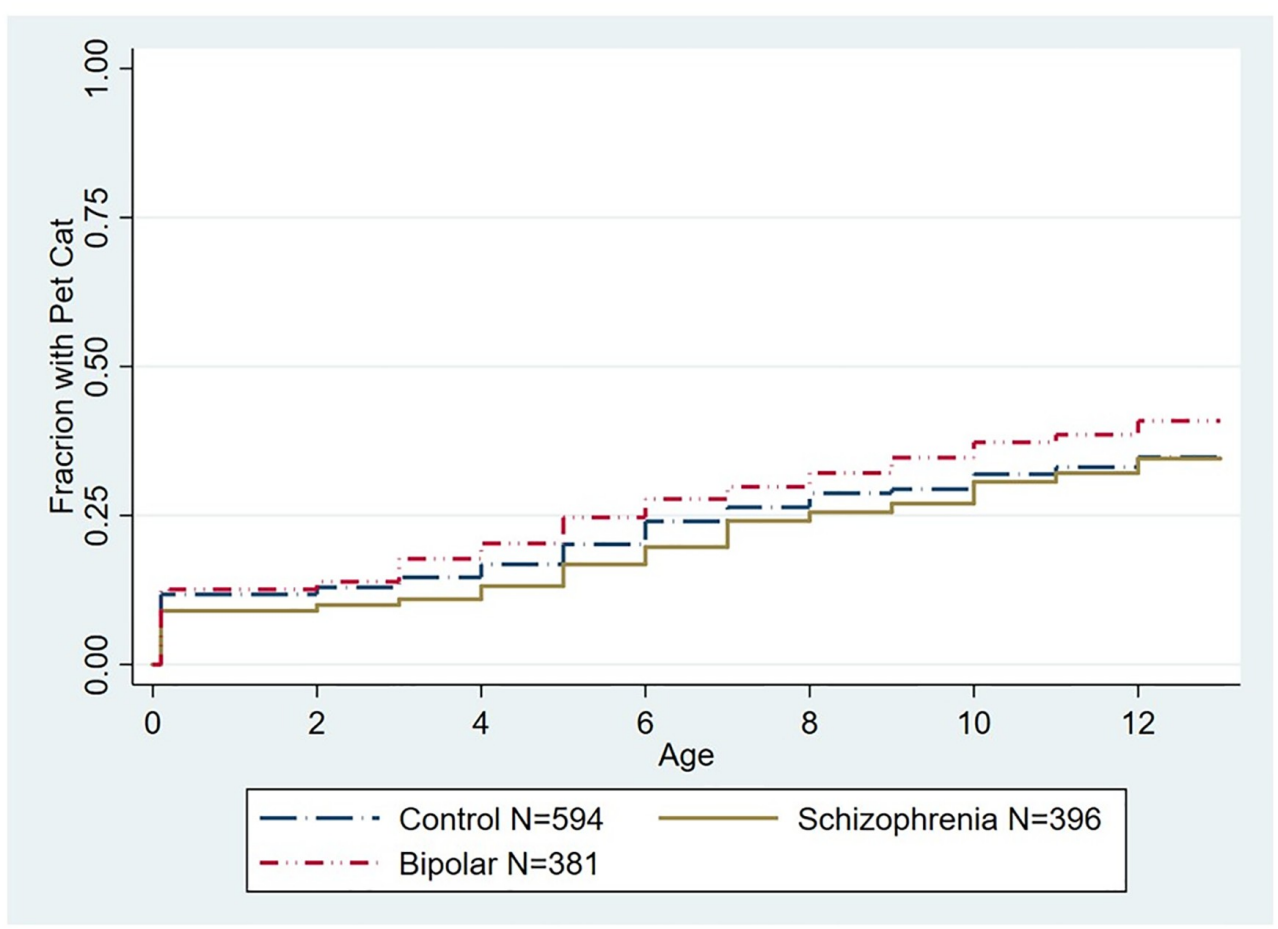
Kaplan Meier plot depicting accumulated proportion of exposure to first household pet cat stratified by diagnostic group. The differences among the groups was not statistically significant (p < .1).

**Fig 4:**
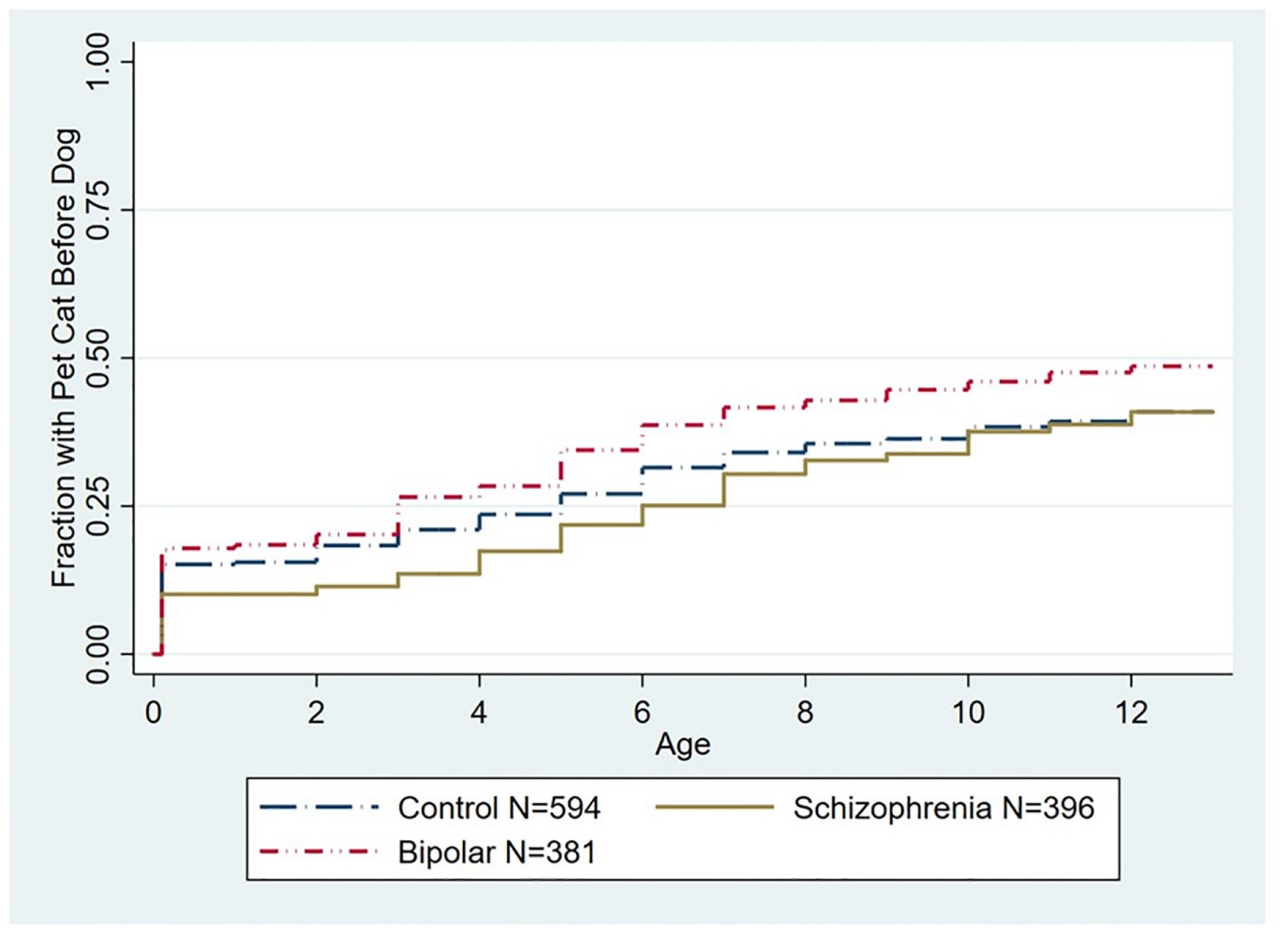
Kaplan-Meier plot depicting accumulated proportion of exposure to first household pet cat stratified by diagnostic group censored for prior exposure to a household pet dog. The age distribution of the censored population is shown in S2 Table. The differences among the groups was not statistically significant (p < .1). The numbers shown indicate the total population including those who were censored.

As depicted in Fig 3, exposure to a household pet cat before the 13th birthday was not associated with a significantly altered hazard of a diagnosis of schizophrenia (HR 1.13, 95% CI .90–1.43, p < .1) or bipolar disorder (HR 1.13, 95% CI .91–1.39, p>.1 both adjusted for age, gender, race/ethnicity, parental education, and place of birth). Exposure to a pet cat was also not associated with an altered hazard of schizophrenia (HR 1.00, 95% CI .80–1.27, p < .1) or bipolar disorder (HR 1.10, 95% CI .89–1.34, p < .1 both adjusted for age, gender, race/ethnicity, parental education, and place of birth) when the population was censored for prior exposure to a household pet dog (Fig 4).

**Fig 5:**
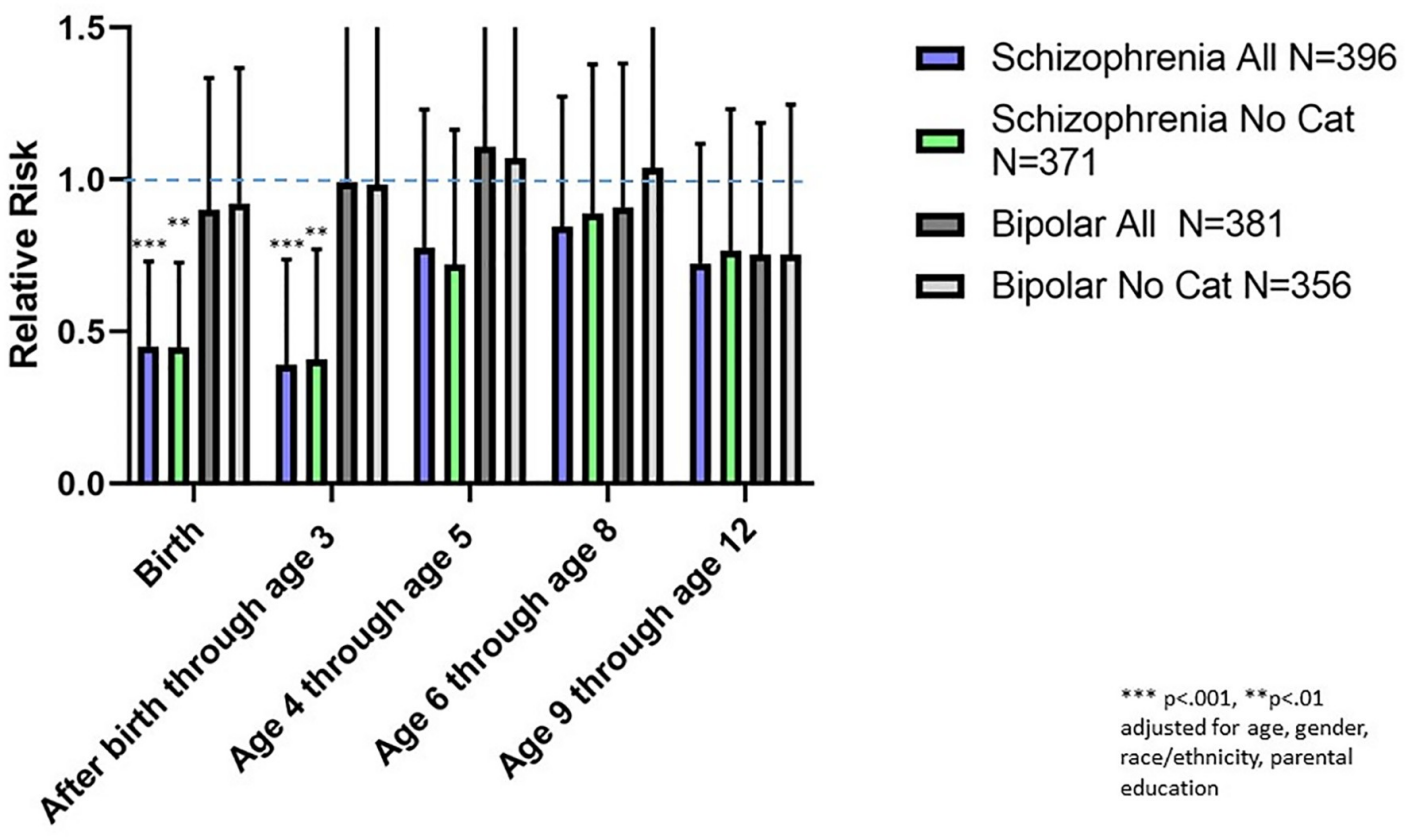
Mean and 95% confidence intervals of relative risks associated with the first household pet dog in the indicated age groups. The relative risks were calculated using multivariate regression models employing age, gender, race, maternal education, and place of birth as covariates. Data are shown for the entire population and the population which did not have prior household exposure to a pet cat.

We employed multinomial logistic regression models to further examine the association between timing of exposure to first pet cat or dog and a subsequent diagnosis of schizophrenia or bipolar disorder. As shown in Fig 5, first exposure to a household pet dog at birth or first exposure during the period between birth and age 3 were associated with a decreased relative risk of a subsequent diagnosis of schizophrenia (exposure at birth: relative risk (RR) .45. 95%CI .27 - .72 p < .001; exposure after birth through age 3, RR .37 95% CI .20 - .70 p < .002 both adjusted for age, gender, race/ethnicity, parental education and place of birth). A similar reduction in relative risk of a schizophrenia diagnosis associated with dog exposure during these age groups was also noted in the subset of individuals previously exposed to a household pet cat (exposure at birth: RR .44, 95% CI .27 -.72 p < .001; exposure after birth through age 3 (RR .38, 95% CI .20 -.74 p < .004 both adjusted for age, gender, race/ethnicity parental education). Exposure to a pet dog at the age ranges 4–5, 6–8, and 9–12 years inclusive were not associated with a significantly altered relative risk of a schizophrenia diagnosis. Exposure to a pet dog was not associated with a significantly altered risk of having a bipolar disorder diagnosis for any of the age groups analyzed (Fig 5).

**Fig 6:**
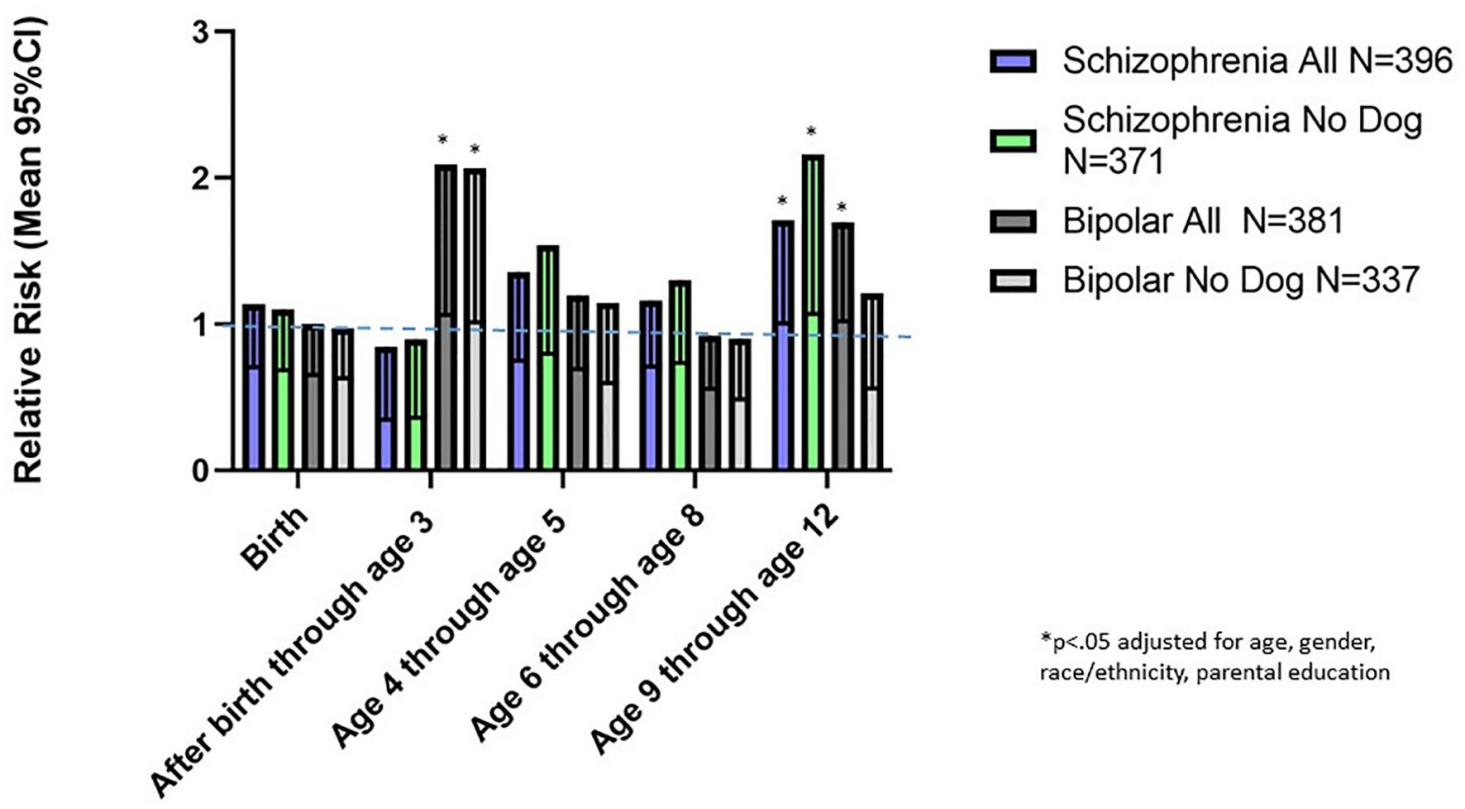
Mean and 95% confidence intervals of relative risks associated with the first household pet cat in the indicated age groups. The relative risks were calculated using multivariate regression models employing age, gender, race, maternal education, and place of birth as covariates. Data are shown for the entire population and the population which did not have prior household exposure to a pet dog.

The associations between the age group of exposure to a first pet cat and subsequent relative risk of a schizophrenia or bipolar disorder diagnosis are shown in Fig 6. There were no statistically significant associations between age group of first cat exposure and either psychiatric diagnosis employing a significance level which takes into consideration multiple comparisons. However, there was a trend towards an increased risk of a schizophrenia diagnosis in individuals first exposed to a pet cat between the ages of 9 and 12. This was evident for both the entire population (RR 1.75. 95% CI 1.02–2.98 p = 0.040) as well as the population censored for previous exposure to a pet dog (RR 2.18, 95% CI 1.08–4.42 p = 0.031, both adjusted for age, gender, race/ethnicity, parental education, and place of birth). There was also a trend toward an increased risk of a bipolar disorder diagnosis and first exposure to a pet cat from the period after birth through age 3 (RR 2.18, 95% CI 1.10–4.31, p < .026). This trend was also detected in the population which had not had a prior exposure to a household pet dog (RR 2.17, 95% CI 1.06–4.46, p < .035 both adjusted for age, gender, race/ethnicity, parental education, and place of birth), There was also a trend towards an association between exposure to a pet cat and a diagnosis of bipolar disorder in the overall population of individuals first exposed to a household pet cat between ages 9 and 12; however this trend was not evident in the population of individuals censored for previous exposure to a pet dog (Fig 6).

## Discussion

We examined the effects of first exposure to a household pet dog and a household pet cat in infancy and childhood in relation to a diagnosis of schizophrenia or bipolar disorder later in life. Our most robust findings were those related to household pet dog exposure. Cox proportional hazard analysis indicated that exposure to a pet dog during the first 12 years of life was associated with an approximately 25% decreased hazard of having a subsequent schizophrenia diagnosis (Fig 1). This association was not explained by a range of demographic factors which may affect household pet exposure including age at evaluation, gender, race, place of birth, and level of parental education. Furthermore, the overall hazard was not altered by prior exposure to a household pet cat (Fig 2). Examination of the age group of first exposure indicated that the apparent protective effect of exposure to a household pet dog was most evident when the household pet dog was present at birth or was added to the household before the end of the second year of life, with exposure during these time points being associated with an approximately 50% reduction in relative risk of a schizophrenia diagnosis (Fig 5). The effect of exposure to a pet dog during different age groups was not affected by prior exposure to a pet cat (Fig 6). Exposure to a pet dog during the period of study did not have a significant effect on the hazard of having a diagnosis of bipolar disorder.

The association between exposure to dogs and subsequent risk of schizophrenia or bipolar disorder has not been extensively studied. One previous investigation based on survey methods reported a trend towards a lower rate of individuals with a diagnosis of schizophrenia, bipolar disorder or schizoaffective disorder whose mothers were exposed to a pet dog during their pregnancy (OR = .71, 95% CI .50 - .99, p = .0427)[13]. Exposure to a household pet dog was associated with a lower rate of depression and anxiety in children aged 4–7. [14] Small studies have reported that therapeutic exposure to a dog can improve symptoms in some individuals with schizophrenia [15]. Two previous studies did not find statistically significant associations between exposure to a pet dog in childhood and a psychiatric disorder but employed relatively small samples of individuals with dog contact [16, 17].

There are several plausible explanations for our finding of a decreased hazard of schizophrenia in individuals exposed to household pet dogs in early life. One possible explanation is that both dog exposure in early life and risk of schizophrenia are associated with demographic factors not measured in our analyses or included in our statistical models. For example, previous studies have shown that having a family pet dog varies by socioeconomic status and a number of geographic factors.[18, 19] Thus, while we partially controlled for socioeconomic status by the measurement of parental education and general location of birth, it is possible that more detailed information regarding these factors would identify covariates which could partially or completely explain the measured associations. Additional sociodemographic factors which can affect pet selection include birth order, family size, contact with farm animals, exposure to feral cats and other animals, and pre-existing allergic or other disorders, data which were not available in our study. We also did not have data relating to the type or breed of dog, which can also vary by geographic and socioeconomic factors.

However, it is also possible that exposure to a pet dog has a direct biological effect on the subsequent hazard of developing schizophrenia. While there are a number of biological mechanisms which might mediate such an interaction, the ones which are most plausible relate to the immune system[20]. Several studies have documented that immune activation during pregnancy and early life as evidenced by exposure to infectious agents and medication increases the risk of developing subsequent psychiatric disorders [21]. Conversely, exposure to a pet dog during pregnancy, infancy, or childhood has been associated with an attenuation of inflammation and a decreased rate of immune mediated disorders such as asthma and food allergies [22, 23], findings which are consistent with what has been characterized as the “hygiene hypothesis”[24] One mechanism by means of which exposure to a pet dog may alter immune activation and risk of immune disorders is the attenuation of the stress response and subsequent cortisol release and cytokine generation in response to antigenic stimuli [25]. It has also been shown that exposure to a household pet dog can alter the intestinal microbiome of family members through contact with canine microflora, suggesting that exposure to pet dogs might affect intestinal inflammation and modulate the risk of schizophrenia through changes in the brain-immune-gut axis [26] or the psycho-immune-neuroendocrine network [27].

It is of note that we found that the lower rate of schizophrenia diagnosis was associated with having a pet dog present in the household at birth, suggesting in utero exposure during pregnancy, or during the first 3 years of life, suggesting exposure during infancy. These time periods correspond to the time periods during which immune activation has been associated with altered neurodevelopment and an increased risk of psychiatric disorders [28].

It is also of note that we did not find an association between exposure to a household pet dog and the hazard of a diagnosis of bipolar disorder. The reason for this difference is not known with certainty but may be the result of differences between the disorders in terms of socio-demographic, immunological, or genetic factors.

In contrast to our findings with pet dogs, proportional hazard models did not identify a significant overall relationship between exposure to a household pet cat in the first 12 years of life and an altered risk of either schizophrenia or bipolar disorder. Significant effects of household pet cat exposure were also not noted in the individuals who had not been previously exposed to a household pet dog. However, while we did not find an overall significant association between exposure to a household pet cat and either schizophrenia or bipolar disorder, we did identify trends toward an association at specific time periods. These included a trend towards an increased relative risk associated with first exposure to a household pet cat between the ages of 9 and 12 for both schizophrenia and bipolar disorder although the trend towards an association between exposure to a household pet cat and risk of bipolar disorder was not evident when individuals previously exposed to a household pet dog were censored from the analysis (Fig 6). An additional trend was identified in terms of an increased relative risk of bipolar disorder in individuals exposed to a household pet cat after birth through the second year of life (RR 2.18, 95% CI 1.10–4.30 p = .026).

The relationship between household exposure to a pet cat during infancy and childhood and subsequent schizophrenia has been the focus of a number of studies with some finding significant associations [29] [30] [31] and others not [32]. Furthermore a large population based study performed in Finland found that exposure to a pet cat in childhood was associated with an increased rate of adult schizotypal traits such as social anhedonia [32] and an increased rate of schizotypal traits was also found in adults with a history of childhood cat bites [33]. However, cat exposure was not significantly associated with psychotic experiences in a population of adolescents following adjustments for covariates [34]. The reasons for the different results among studies of pet cat exposure are not known with certainty but are likely the effects of different social, demographic and biological variables associated with exposure. Our findings also suggest that the age of first pet cat exposure may be an important variable in terms of disease risk as is evident in terms of pet dog exposure. It is of note that we detected a trend towards an increased risk of a schizophrenia diagnosis in individuals first exposed to a pet cat between the ages of 9 and 12 irrespective of prior exposure to a pet dog. Additional studies will be important to determine if this association is evident in other populations. It is important that future studies include more detailed information in terms of the timing of exposure in order to better define the risks and benefits of pet exposure in early life.

There were a number of strengths to our study. These include the relatively large sample size of individuals with defined psychiatric disorders and the use of identical methods of data collection and ascertainment of pet exposure for individuals with schizophrenia and bipolar disorder as well as controls. Furthermore, identical methods were used to collect data on household exposure to pet cats and pet dogs minimizing the likelihood of bias.

There were also a number of limitations of our study. As noted above there were a number of socio-demographic factors which might be relevant to pet exposure and subsequent risk of psychiatric disorders. In addition, exposure to a household pet during childhood was based on self-report by individuals who were adults when they were queried. Previous studies have demonstrated that adults have a fairly high (~80%) accuracy of recall of pet exposure in childhood although there is some degree of underreporting.[35] While the reliance on recall in our study may have led to some degree of bias in our study, the self-report of history having a household pet cat or dog had different effects on the subsequent development of schizophrenia suggesting that any recall bias would need to be differential to have a confounding effect. While persons with schizophrenia may be subject to deficits in autobiographical memory, there is no reason to suspect that their recall would be differential for pet dogs as compared to pet cats [36]. Another limitation of our study is that the case and control groups are selected in terms of their willingness to participate and thus may not be totally representative of the population of individuals who do and do not have a psychiatric disorder. Furthermore, available data on socioeconomic status was limited to the measure of parental education. While widely used as a proxy for socioeconomic status [37], more sophisticated measures such as those involving geocoding [38] would provide more detailed information. Another limitation involves the lack of a complete set of data relating to other health measures such as body mass index which might modulate the association between animal exposure, socioeconomic status, and risk of psychiatric disorders [39].

### Conclusion

In our study population, exposure to a household pet dog at birth and during the first three years of life is associated with a significantly decreased hazard and relative risk of a subsequent diagnosis of schizophrenia. Trends in associations between childhood household exposure to a pet cat and relative risks of schizophrenia and bipolar disorder were also detected. These associations may be due to socio-demographic, neuro-immune, or other biological factors or combinations of factors. An understanding of the mechanisms underlying these associations could provide insights into the role of environmental exposures as risk factors for these disorders and inform appropriate interventions.

## Supporting information

S1 Table Age of first cat exposure without prior exposure to a pet dog. (DOCX)

S2 Table Age of first dog exposure without prior exposure to a pet cat. (DOCX)

S3 Table Detailed description of the individual data employed for the statistical analyses. The categories of age and place of birth have been broadened to ensure participant confidentiality. Abbreviations employed: Ctr = Unaffected Control, Scz = Schizophrenia, BP Bipolar Disorder.
(XLSX)
