# Supplementary material for: Exposure to household pet cats and dogs in childhood and risk of subsequent diagnosis of schizophrenia or bipolar disorder
Source: PLoS One. 2019 Dec 2;14(12):e0225320. doi: 10.1371/journal.pone.0225320 (PMC6886852; doi:10.1371/journal.pone.0225320)
Supplement: S1 Table — (DOCX) [file pone.0225320.s001.docx]

**S1 Table. Age of First Cat exposure without Prior Exposure to a Pet Dog.**

| Age of First Pet Cat Without a Prior Dog |  |  |  |  |
| --- | --- | --- | --- | --- |
|  |  | Schizophrenia | Bipolar Disorder | Control |
| Total (Censored for Prior Exposure) |  | 371 | 337 | 547 |
|  |  |  |  |  |
|  |  |  |  |  |
| Present at Birth | Number | 37 | 47 | 70 |
|  | % | 9.97 | 13.95 | 12.8 |
| After Birth through Age 3 | Number | 8 | 19 | 15 |
|  | % | 2.16 | 5.64 | 2.74 |
| Age 4-5 | Number | 21 | 18 | 23 |
|  | % | 5.66 | 5.34 | 4.2 |
| Age 6-8 | Number | 27 | 17 | 33 |
|  | % | 7.28 | 5.04 | 6.03 |
| Age 9-12 | Number | 21 | 12 | 18 |
|  | % | 5.66 | 3.56 | 3.29 |
| Any Before Age 13 | Number | 114 | 113 | 159 |
|  | % | 30.73 | 33.53 | 29.07 |
| None Before Age 13 | Number | 257 | 224 | 388 |
|  | % | 69.27 | 66.47 | 70.93 |
