# Supplementary material for: Exposure to household pet cats and dogs in childhood and risk of subsequent diagnosis of schizophrenia or bipolar disorder
Source: PLoS One. 2019 Dec 2;14(12):e0225320. doi: 10.1371/journal.pone.0225320 (PMC6886852; doi:10.1371/journal.pone.0225320)
Supplement: S2 Table — (DOCX) [file pone.0225320.s002.docx]

**S2 Table. Age of First Dog Exposure without Prior Exposure to a Pet Cat**

| Age of First Pet Dog Without a Prior Pet Cat |  |  |  |  |
| --- | --- | --- | --- | --- |
|  |  | Schizophrenia | Bipolar Disorder | Control |
| Total (Censored for Prior Cat Exposure) |  | 371 | 356 | 549 |
|  |  |  |  |  |
| Present at Birth | Number | 32 | 71 | 100 |
|  | % | 8.63 | 19.94 | 18.21 |
| After Birth through Age 3 | Number | 17 | 31 | 43 |
|  | % | 4.58 | 8.71 | 7.83 |
| Age 4-5 | Number | 43 | 44 | 57 |
|  | % | 11.59 | 12.36 | 10.38 |
| Age 6-8 | Number | 59 | 47 | 64 |
|  | % | 15.9 | 13.2 | 11.66 |
| Age 9-12 | Number | 44 | 30 | 60 |
|  | % | 11.86 | 8.43 | 10.93 |
| Any Before Age 13 | Number | 195 | 223 | 324 |
|  | % | 52.56 | 62.64 | 59.01 |
| None Before Age 13 | Number | 176 | 133 | 225 |
|  | % | 47.44 | 37.36 | 40.98 |
